# Angiotensin-converting enzyme inhibition and food restriction in diabetic mice do not correct the increased sensitivity for ischemia-reperfusion injury

**DOI:** 10.1186/1475-2840-11-89

**Published:** 2012-08-01

**Authors:** Gerry Van der Mieren, Ines Nevelsteen, Annelies Vanderper, Wouter Oosterlinck, Willem Flameng, Paul Herijgers

**Affiliations:** 1 Department of Cardiovascular Sciences, Research Unit Experimental Cardiac Surgery, K.U. Leuven, Herestraat 49, B-3000, Leuven, Belgium

**Keywords:** Ischemia/reperfusion, Diabetes mellitus, Metabolic syndrome, In vivo contractility, Infarct size

## Abstract

**Background:**

The number of patients with diabetes or the metabolic syndrome reaches epidemic proportions. On top of their diabetic cardiomyopathy, these patients experience frequent and severe cardiac ischemia-reperfusion (IR) insults, which further aggravate their degree of heart failure. Food restriction and angiotensin-converting enzyme inhibition (ACE-I) are standard therapies in these patients but the effects on cardiac IR injury have never been investigated. In this study, we tested the hypothesis that 1° food restriction and 2° ACE-I reduce infarct size and preserve cardiac contractility after IR injury in mouse models of diabetes and the metabolic syndrome.

**Methods:**

C57Bl6/J wild type (WT) mice, leptin deficient ob/ob (model for type II diabetes) and double knock-out (LDLR-/-;ob/ob, further called DKO) mice with combined leptin and LDL-receptor deficiency (model for metabolic syndrome) were used. The effects of 12 weeks food restriction or ACE-I on infarct size and load-independent left ventricular contractility after 30 min regional cardiac ischemia were investigated. Differences between groups were analyzed for statistical significance by Student’s t-test or factorial ANOVA followed by a Fisher’s LSD post hoc test.

**Results:**

Infarct size was larger in ob/ob and DKO versus WT. Twelve weeks of ACE-I improved pre-ischemic left ventricular contractility in ob/ob and DKO. Twelve weeks of food restriction, with a weight reduction of 35-40%, or ACE-I did not reduce the effect of IR.

**Conclusion:**

ACE-I and food restriction do not correct the increased sensitivity for cardiac IR-injury in mouse models of type II diabetes and the metabolic syndrome.

## Background

The number of patients with diabetes and the metabolic syndrome increases in Western societies and reaches epidemic proportions [1,2]. At present, diabetes affects approximately 250 million people worldwide and by 2025 this is expected to increase to over 380 million, with type II diabetes accounting for 90-95% of them. Prevalence is expected to increase most in Asia and Africa with the majority of patients in 2030 being found there [2]. The prevalence of the metabolic syndrome currently exceeds 20% of individuals who are over 20 years of age and 40% of the population older than 40 years [1]. Heart failure is the leading cause of mortality in people with type II diabetes. The incidence of myocardial infarction in diabetic patients is twice that of the general population [3,4]. They are at increased risk for mortality and post-ischemic complications [3,4]. Infarct size for a given ischemic insult is larger in diabetic mice than in controls [5-7]. This, on top of diabetic cardiomyopathy [8,9], contributes to progressive heart failure. Therefore, new or additional techniques to protect the diabetic heart against this ischemia/reperfusion (IR) damage are eagerly awaited.

Food restriction and ACE-inhibition (ACE-I) are frequently used therapies in type II diabetes [10].

Although food restriction is a standard therapy in type II diabetic patients, no long-term large-scale study of intentional weight loss has been adequately powered to examine cardiovascular disease end points in patients with diabetes [10]. Conflicting data were reported concerning the effect of food restriction on cardiac contractility in experimental models. Some studies in diabetic mice report a reduced mean 24-hour blood pressure and heart rate, restored circadian variations of blood pressure and heart rate, and increased ejection fraction [11]. In other studies with obese rats, stroke volume, left ventricular work and cardiac output decreased significantly after food restriction [12]. Similarly, the effect of food restriction on the impact of IR injury was barely investigated. In a study with wild type rats, ex vivo cardiac contractility was better preserved after IR injury after 8-months of food restriction [13]. The effect of food restriction on IR injury in diabetic or metabolic syndrome models was never investigated.

Current guidelines advocate ACE-inhibitors as the drugs of choice in the initial treatment of hypertension in diabetic patients [10]. ACE-I is favored because, in addition to the blood pressure lowering properties, it has well documented anti-ischemic and anti-atherogenic effects, reduces oxyradical formation and has an effect on cardiovascular remodeling. ACE-I improves glucose control and insulin sensitivity and slows the progression of diabetic nephropathy [14]. In a large meta-analysis of patients with acute myocardial infarction, ACE-I reduced 30-day mortality from 7.6 to 7.1% and in the subgroup of diabetic patients from 12.0 to 10.3% [15].

The effect of ACE-I in experimental models of acute IR injury is uncertain [16-21]. Experiments with captopril in a canine, porcine and rat model report a reduced myocardial injury although other experiments in canine models do not show any effect [16,17]. Studies with other ACE-inhibitors report also heterogeneous data. Some report a reduction in infarct size [18,19] while others see no effect [20]. In Langendorff-perfused mouse hearts, captopril has no beneficial effect on post-ischemic contractile recovery or cardiac enzyme release [21]. Nevertheless, when cardiac p90 ribosomal S6 kinase is over-expressed in mice, captopril induces a better post-ischemic contractility and reduced enzyme release [21]. Since p90 ribosomal S6 kinase is activated in hyperglycemic mice, it might be possible that captopril induces protection in diabetic mice models.

Therefore, the aim of this study is to investigate the effects of food restriction and ACE-I on IR injury in diabetic or metabolic syndrome models. We tested the hypotheses that 1° food restriction and 2° ACE-I do correct the increased sensitivity for cardiac IR injury.

## Methods

### Animal models

Experiments were conducted in C57BL/6J WT-mice, leptin deficient ob/ob, and double knock-out (DKO) mice with combined leptin and LDL-receptor deficiency (total number = 145). The ob/ob mouse is a model for type II diabetes, featuring abdominal obesity and insulin resistance. DKO mice feature many characteristics of the metabolic syndrome, i.e. obesity, dyslipidemia, hypertension, insulin resistance and impaired glucose tolerance and/or diabetes [8,11]. These models develop left ventricular diastolic and systolic dysfunction comparable with the diabetic cardiomyopathy seen in patients [8].

Ob/ob and C57BL/6J mice were purchased from Jackson Laboratory (Bar Harbor, Maine, USA). DKO were generated as described previously [8,11]. IR was induced at 24 weeks of age. The investigation conforms with the Guide for the Care and Use of Laboratory Animals published by the US National Institutes of Health (NIH Publication 1996). All experimental protocols were approved by the K.U.Leuven Institutional Animal Care Commission and Ethical Committee.

### Treatments

Food intake of diet-restricted ob/ob and DKO mice was restricted to 2.5 g/day, which is the normal daily intake of lean WT mice, between 12 and 24 weeks of age [11].ACE-inhibition was obtained with captopril (10 mg/kg/day) intraperitoneally from 12 until 24 weeks of age in WT, ob/ob and DKO [22].

### Biochemical analysis

Blood of conscious mice was collected by tail bleeding into EDTA tubes after a 24 h fast at 12 and 24 weeks of age. Plasma was obtained by centrifugation. Triglycerides and total cholesterol was determined with a diagnostic reagent kit (Roche) and glucose with a glucometer (Menarini Diagnostics).

### Ischemia/reperfusion

The experimental technique was previously described [23]. Briefly, anesthesia was induced with urethane (1.2 g/kg) and alfa-chloralose (50 mg/kg). Mice were ventilated with room air, with rectal temperature kept at 37 ± 0.5°C. Via left thoracotomy, the left anterior descending artery (LAD), was non-traumatically occluded, 2 mm below the tip of the left auricle for 30 min. Afterwards, a reperfusion period of 1 hour was allowed. Successful coronary occlusion and reperfusion was visually verified by observing the myocardium distal to the coronary occlusion turning pale respectively blushing. In the groups without ischemia (sham), a thoracotomy and time-matched procedure was performed.

### Outcome parameters: infarct size and in vivo left ventricular contractility

The technique was previously described [23]. At the end of 1 hour reperfusion, a pressure-conductance catheter (1.4-Fr, SPR-839; Millar Instruments, Houston, TX) was inserted through the right carotid artery into the left ventricle. Baseline pressure-volume (PV) loops were recorded (Powerlab/AD Instruments, Castle Hill, Australia). Parallel volume and specific blood conductance were determined [8,23]. The inferior caval vein was compressed to obtain left ventricular PV-loops under varying loading conditions. Heart rate, systolic and end-diastolic pressure were measured. Stroke volume was determined as the difference in end-diastolic and end-systolic volume. Stroke work (SW) is the mechanical energy which the heart develops during the cardiac cycle and is calculated as the area enclosed by the PV-loop.

Preload recruitable stroke work (PRSW) is the slope of the relationship between end-diastolic volume and SW performed by the ventricle. PRSW is the most reliable and useful parameter for general contractility since it is chamber size independent and robust [24]. The slope of the end-systolic pressure-volume relationship, end-systolic elastance (E_es_), reflects left ventricular chamber end-systolic stiffness and is used as an index of contractility. Tau is the time constant of left ventricular relaxation during isovolumetric diastole. The end-diastolic PV-relationship (EDPVR) represents the compliance of the ventricular myocardium at the end of the diastole. Augmented stiffness of the ventricular wall increases the slope of the EDPVR. Arterial elastance (E_a_), a measure for afterload, is defined as the end-systolic pressure to stroke volume ratio [23,24].

Before excision of the heart, Evans blue (0.8 ml, 1% solution) was injected intravenously after re-occlusion of the LAD, to determine the left ventricular perfusion area at risk. The heart was cut in 1 mm-slices and the slices were stained with triphenyl-tetrazolium-chloride solution (TTC, 20 min, 1%, 37°C, pH 7.4) and fixed in paraformaldehyde (10 min, 4% solution, 20°C). All slices were weighed and photographed with a digital camera under magnification.

### Data management and statistical analysis

Analysis of the pressure-conductance data was performed using PVAN 3.2 software (Millar Instruments, Houston) as previously described [8,23].

Infarct size and area at risk were determined by the number of pixels in each zone with Adobe Photoshop 8.0 (Adobe System Inc.) multiplied by the weight of the respective slices.

All statistical analyses were performed using Statistical software (Statistica 7.1, StatSoft, Tulsa, USA). Data are expressed as mean ± standard deviation. Differences between groups were analyzed for statistical significance by Student’s t-test or factorial ANOVA followed by a Fisher’s LSD post hoc test. A value of p < 0.05 was considered significant.

## Results

### Biochemical parameters and contractility in untreated mice

**Figure 1:**
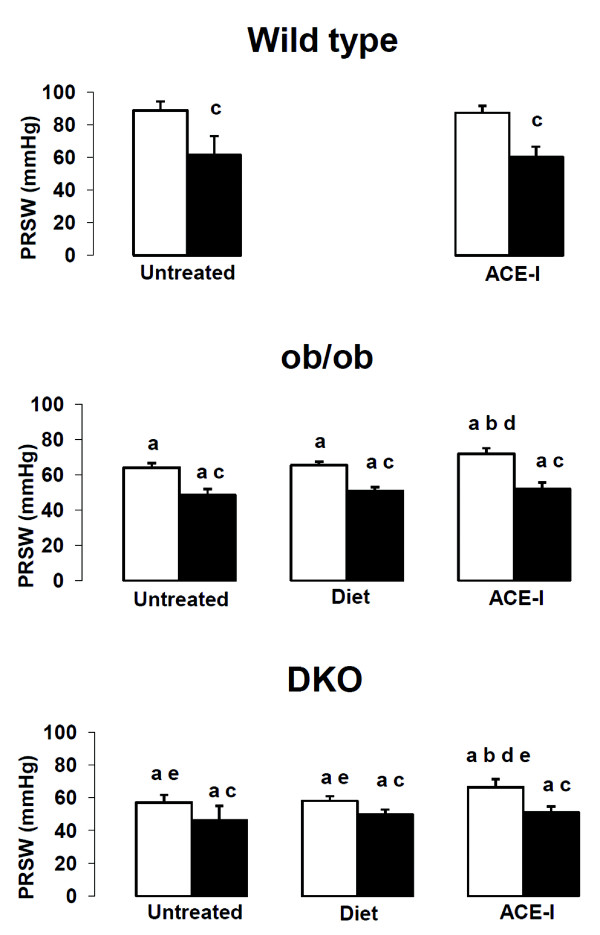
**Preload recruitable stroke work in wild type, ob/ob and double knock-out mice**. ^“a”^ p < 0.05 versus WT same treatment, same age; ^“b”^ p < 0.05 versus same untreated genotype, same age; ^“c”^ p < 0.05 versus sham, same genotype, same treatment; ^“d”^ p < 0.05 versus diet, same genotype, same age^“e”^ p < 0.05 versus ob/ob same treatment, same age.

Untreated ob/ob and DKO have a significantly higher weight and exhibit hyperglycemia (Table1). Cholesterol levels are elevated in ob/ob and even higher in DKO. DKO also have increased triglycerides levels. Left ventricular contractility, expressed as PRSW, is worse in ob/ob than in WT and even worse in DKO (Figure1). Diastolic function, represented by end-diastolic pressure and EDPVR, is significantly worse in DKO than in WT (Table2). Ejection fraction is not reported since it is highly dependent upon preload and afterload, leading to significant misinterpretations of contractility in diabetic mice [9]. 

**Table 1:** Metabolic parameters

**Genotype**	**Age (weeks)**	**Treatment**	**Weight (g)**	**Heart weight / tibial lenght (mg/cm)**	**Glucose (mg/dl)**	**Cholesterol (mg/dl)**	**Triglycerides (mg/dl)**
Wild type	12	Untreated	21.1 ± 3.1		56.2 ± 6.5	104 ± 70	115 ± 42
	24	Untreated	28.5 ± 4.4	74.1 ± 9.0	71.2 ± 11.8	76 ± 14	60 ± 14
Ob/ob	12	Untreated	49.5 ± 3.5^a^		163 ± 73^a^	125 ± 20	58 ± 11^a^
	24	Untreated	62.7 ± 3.7^a^	76.6 ± 7.7	166 ± 41^a^	139 ± 35^a^	68 ± 12
	24	Diet	38.5 ± 4.4^ac^	71.8 ± 7.1^c^	142 ± 63^a^	74 ± 24^c^	69 ± 15
	24	ACE-I	64.1 ± 9.4^ad^	74.8 ± 9.1	84 ± 19^acd^	145 ± 34^ad^	76 ± 13^a^
DKO	12	Untreated	47.0 ± 2.4^a^		155 ± 47^a^	912 ± 188^ab^	591 ± 181^ab^
	24	Untreated	59.4 ± 9.3^a^	79.3 ± 13.4	152 ± 108^a^	749 ± 114^ab^	245 ± 80^ab^
	24	Diet	38.2 ± 3.6^ac^	68.0 ± 8.5^ac^	141 ± 62^a^	754 ± 123^ab^	252 ± 110^ab^
	24	ACE-I	64.0 ± 8.0^ad^	70.3 ± 7.2^c^	124 ± 36^ab^	658 ± 166^ab^	202 ± 67^ab^

^“a”^ p < 0.05 versus WT, same age, untreated; ^“b”^p < 0.05 versus ob/ob, same age, same treatment; ^“c”^ p < 0.05 versus same untreated genotype , same age; ^“d”^ p < 0.05 versus diet, same genotype, same age.

**Table 2:** Hemodynamic parameters

	***Condition***	**Wild type**		**Ob/ob**		**DKO**	
		***Sham***	***IR***	***Sham***	***IR***	***Sham***	***IR***
**Heart rate (bpm)**	Untreated	575 ± 40	560±62	526 ± 44^a^	540±41	523 ± 29^a^	513±38
	Diet			504 ± 30^a^	500 ± 31	503 ± 34^a^	516±51
	ACE-I	548 ± 33	524 ± 36	505 ± 29	492±33	499 ± 51	509±68
**Stroke volume (μl)**	Untreated	16.2 ± 5.3	6.8±3.2^c^	14.8 ± 3.1	12.0±4.3^a^	10.1 ± 5.1^a^	9.6±4.1
	Diet			13.6 ± 6.0	14.2±7.0^a^	11.8 ± 5.1	12.7±6.1^a^
	ACE-I	16.5 ± 4.9	8.1 ± 1.6^c^	14.0 ± 3.2	12.6±4.2^a^	15.1 ± 6.1	12.4±3.0^a^
Stroke work (mmHg*μl)	Untreated	1256 ± 328	263±167^c^	835 ± 189^a^	509±181^ac^	606 ± 269^a^	409±199
	Diet			804 ± 546	667±336^a^	627 ± 272^a^	676±332^a^
	ACE-I	877 ± 399	369 ± 158^c^	788 ± 251	581±230	780 ± 408	563±170
**P**_**sys**_**(mmHg)**	Untreated	80.5 ± 18.2	55.1±10.6^c^	68.7 ± 7.5	62.5±2.7	69.5 ± 11.1	62.7±10.6
	Diet			68.4 ± 10.8	60.4±5.9	67.1 ± 12.6	65.0±5.5^a^
	ACE-I	62.9 ± 11.9	61.9 ± 19.9	67.9 ± 6.9	59.2±7.5^c^	61.4 ± 6.2	56.1±8.1^d^
**P**_**ed**_**(mmHg)**	Untreated	3.0 ± 0.7	2.9±1.2	3.6 ± 0.7	6.6±3.3^ac^	4.8 ± 2.3^a^	6.6±2.4^a^
	Diet			3.0 ± 1.1	4.3±1.2^a^	3.8 ± 1.3	4.3±1.5^ab^
	ACE-I	3.6 ± 2.0	2.9 ± 2.3	4.5 ± 0.9^d^	5.0±1.5	3.7 ± 0.9	3.4±1.5^b^
**PRSW (mmHg)**	Untreated	88.8 ± 5.5	61.5 ± 11.6^c^	64 ± 2.6^a^	48.5 ± 3.3^ac^	57.1 ± 4.6^ae^	46.2 ± 8.8^ac^
	Diet			65.4 ± 1.9^a^	51 ± 1.9^ac^	58 ± 2.8^ae^	49.7 ± 3^ac^
	ACE-I	87.4 ± 4.4	60.2 ± 6.4^c^	71.8 ± 3.2^abd^	52.1 ± 3.4^ac^	66.3 ± 5^abde^	51.2 ± 3.4^ac^
**E**_**es**_**(mmHg/μl)**	Untreated	8.3 ± 2.5	6.4±1.5	5.1 ± 0.4^a^	4.7±1.5	6.5 ± 2.4	4.5±0.8^a^
	Diet			6.6 ± 2.1	4.5±1.1^ac^	5.1 ± 1.2^a^	5.3±1.2
	ACE-I	6.0 ± 1.0	6.8 ± 2.0	5.5 ± 1.7	4.6±1.7	5.6 ± 1.3	4.4±1.3^a^
**Tau (ms)**	Untreated	6.3 ± 1.2	7.6±2.2	6.2 ± 0.5	6.4±1.0	6.9 ± 0.9	7.2±0.7
	Diet			6.9 ± 0.5^b^	7.7±0.8^bc^	6.8 ± 0.6	7.3±1.2
	ACE-I	6.6 ± 1.5	7.4 ± 1.5	8.0 ± 1.1^bd^	8.8±1.5^b^	7.4 ± 0.8	7.1±1.1^e^
**EDPVR (mmHg/μl)**	Untreated	0.2 ± 0.2	0.4±0.2	0.4 ± 0.3	0.5±0.3	0.6 ± 0.4^a^	0.3±0.1
	Diet			0.2 ± 0.2	0.2±0.1	0.2 ± 0.2	0.2±0.1^ab^
	ACE-I	0.2 ± 0.1	0.4 ± 0.3	0.4 ± 0.1	0.3±0.1	0.3 ± 0.1^be^	0.3±0.1^d^
**E**_**a**_**(mmHg/μl)**	Untreated	4.8 ± 1.5	8.1 ± 2.4^c^	4.0 ± 0.9	5.0±2.0^a^	6.8 ± 3.5	5.6±1.8^a^
	Diet			4.9 ± 1.8	4.8±2.7^a^	5.2 ± 2.2	5.4±2.1^a^
	ACE-I	3.3 ± 0.6^b^	6.9 ± 1.3^c^	4.6 ± 1.5	4.5±1.5^a^	3.4 ± 1.3^b^	3.8±1.0^ab^

ACE-I: angiotensin-converting enzyme inhibition; DKO: double knock-out (ob/ob; LDLR-/-); E_a_: arterial elastance; EDPVR: end-diastolic pressure-volume relationship; E_es_: end-systolic elastance; IR: ischemia/reperfusion; P_ed_: end-diastolic pressure; P_sys_: systolic pressure ^“a”^ p < 0.05 versus WT same treatment, same condition (sham or IR); ^“b”^ p < 0.05 versus same untreated genotype, same condition (sham or IR); ^“c”^ p < 0.05 versus sham, same genotype, same treatment; ^“d”^ p < 0.05 versus diet, same genotype, same condition (sham or IR); ^“e”^ p < 0.05 versus ob/ob same treatment, same condition (sham or IR).

### Effect of IR

**Figure 2:**
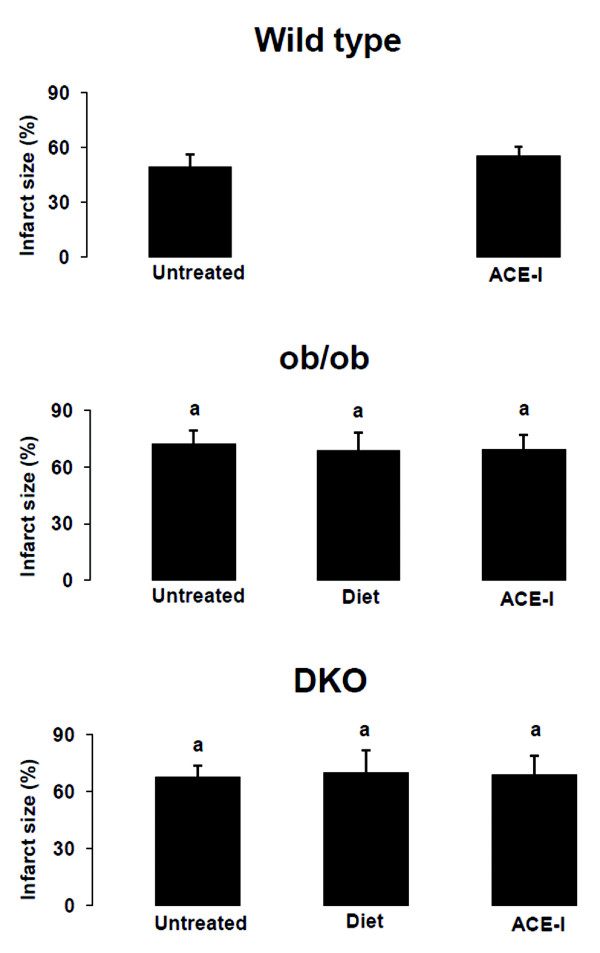
**Infarct size as % of risk zone in wild type, ob/ob and double knock-out mice**^“a”^ p < 0.05 versus WT same treatment, same age; ^“b”^ p < 0.05 versus same untreated genotype, same age; ^“c”^ p < 0.05 versus sham, same genotype, same treatment; ^“d”^ p < 0.05 versus diet, same genotype, same age; ^“e”^ p < 0.05 versus ob/ob same treatment, same age.

The area at risk after coronary occlusion is comparable in all groups (Table3). As expected, none of the sham groups showed an infarct. After IR, infarct size was larger in DKO and ob/ob versus WT (Figure2). After IR, PRSW decreased significantly in all groups and was significantly lower in DKO and ob/ob versus WT (Figure1).

**Table 3:** Numbers and infarct size

	***Condition***	**Wild type**		**Ob/ob**		**DKO**	
		***Sham***	***IR***	***Sham***	***IR***	***Sham***	***IR***
**N experiments (survivors)**	Untreated	9 (9)	12 (10)	7 (7)	11 (6)	7 (7)	9 (7)
	Diet			9 (7)	11 (7)	9 (7)	9 (8)
	ACE-I	6 (6)	8 (6)	10 (8)	10 (8)	8 (8)	10 (8)
**Area at risk (% of heart)**	Untreated	0 ± 0	14.7 ± 7.5	0 ± 0	14.0 ± 6.7	0 ± 0	18.2 ± 7.2
	Diet			0 ± 0	17.8 ± 3.4	0 ± 0	16.4 ± 8.6
	ACE-I	0 ± 0	17.0 ± 7.1	0 ± 0	17.1 ± 3.4	0 ± 0	15.3 ± 5.6
**Infarct size (% of area at risk)**	Untreated	0 ± 0	49.3 ± 6.8^b^	0 ± 0	72.4 ± 7.2^ab^	0 ± 0	67.2 ± 6.1^ab^
	Diet			0 ± 0	68.4 ± 9.8^ab^	0 ± 0	70 ± 11.9^ab^
	ACE-I	0 ± 0	55.1 ± 5.7^b^	0 ± 0	69 ± 8.1^ab^	0 ± 0	68.8 ± 9.9^ab^

ACE-I: angiotensin-converting enzyme inhibition; DKO: double knock-out (ob/ob; LDLR-/-); ^a”^ p < 0.05 versus WT same treatment, same condition (sham or IR); ^“b”^ p < 0.05 versus sham, same genotype, same treatment.

### Effect of food restriction on biochemical parameters and contractility

Food restriction resulted in lower body and heart weight in both ob/ob and DKO (Table1). Heart weight was even lower in food restricted DKO than in WT. Food restriction had little effect on biochemical parameters. Food restriction did not improve the reduced PRSW in ob/ob and DKO mice (Figure1).

### Effect of food restriction on IR injury

Food restriction did not reduce infarct size in DKO nor ob/ob versus the untreated groups (Figure2). The impact of IR on PRSW was not influenced by food restriction (Figure1).

### Effect of ACE-I on biochemical parameters and contractility

ACE-I reduced glycemia levels in ob/ob but much less in DKO (Table1). Heart weight, was lower in DKO but not in ob/ob after ACE-I compared with the untreated. PRSW was significantly better after 12 weeks of ACE-I in ob/ob and DKO versus diet (Figure1, Table2).

### Effect of ACE-I on IR injury

ACE-I did not reduce infarct size in any genotype versus the untreated groups (Figure2). Furthermore, ACE-I did not reduce the impact of IR on PRSW in any genotype (Figure1).

## Discussion

The number of patients with diabetes and the metabolic syndrome reaches epidemic proportions [1,2]. The incidence of myocardial infarction in diabetic patients is twice that of the general population [3,4]. Infarct size for a given ischemic insult is larger in diabetic patients than in non-diabetic patients [3,4]. This, on top of diabetic cardiomyopathy [8,9], contributes to progressive heart failure. Therefore, new or more potent strategies to protect them against cardiovascular complications are needed. Our mice models of type II diabetes and the metabolic syndrome are, similar to the clinical reality, more vulnerable for IR injury. In other experimental diabetic models, disagreement exists about the sensitivity to IR injury [25-28]. Depending on the reports, infarct size in animal models of diabetes was larger, smaller or similar to non-diabetic controls [26]. There are numerous differences in experimental protocols and a single factor cannot entirely explain the discrepancies. Duration of the diabetic state and plasma level of insulin (type I versus type II) seems to influence the myocardial susceptibility to IR injury [26]. Although type II diabetes represents 90% of the diabetic patients, the majority of the experimental studies are performed in type I diabetic rat models, induced by streptozotocin [5,25-27]. Most of them report a similar or reduced myocardial infarct area after IR injury [25,26]. Rodrigues et al. [25] reported that 3 months after IR injury, also ventricular dysfunction was attenuated and the profile of calcium handling proteins was better. Nevertheless, the mortality was higher in the type I diabetic group, due to increased autonomic dysfunction [25]. The sensitivity to IR injury in this model seems to be time-dependent. Some studies report a resistance to IR early after diabetes induction by streptozotocin, which disappears after 2 months [26]. Others however demonstrated an increased infarct size after coronary occlusion as early as 8 days after streptozotocin injection [26], indicating that duration of diabetes is not the only factor responsible for the heterogeneous results. Disagreement also exists about the effect of acute hyperglycemia to IR injury. Although some studies report a larger or similar susceptibility to IR injury in a hyperglycemic state, other reports demonstrate a significant protection, related to a better profile of calcium handling proteins [25,26].

The difference in presence or absence of hyperinsulinemia may also be responsible for the heterogeneous data. With the exceptions of a few studies in type II diabetic Zucker and Goto-Kakizaki rats, all type II diabetic models with obesity and hyperinsulinemia showed increased myocardial susceptibility to IR injury [26]. In the past decade, transgenic mouse models of type II diabetes and the metabolic syndrome have been developed. These models develop left ventricular diastolic and systolic dysfunction comparable with the diabetic cardiomyopathy seen in patients [8]. We demonstrated in this study that these mice models of type II diabetes and the metabolic syndrome are more vulnerable for IR injury than their WT littermates. This correlates well with data from patient studies and previous studies in transgenic mice with type II diabetes [6,7,26]. Nevertheless, the underlying mechanisms by which type II diabetic and metabolic syndrome myocardium is more vulnerable to IR injury remains incompletely understood. Several hypotheses were suggested.

First, the diabetic myocardium exhibits abnormal cardiac substrate utilization and impaired energetic efficiency. In non-ischemic conditions, the myocardium uses preferentially fatty acids as energy substrate. This is even more the case in type II diabetes and the metabolic syndrome. The use of fatty acids requires 12% more oxygen per ATP generated than glucose. During ischemia, the normal myocardium switches to glucose as preferred energy substrate. This possibility is lost in case of diabetes, because oxidation of fatty acids inhibits the catabolism of glucose [29].

Second, endothelial dysfunction and reduced nitric oxide (NO) bioavailability is present in type II diabetes and the metabolic syndrome [11,14]. The beneficial or detrimental effect of NO depends on its concentration and cellular origin [5]. Although constitutive production of NO by endothelial cells (eNOS) is thought to be cytoprotective, NO generated by the inducible form (iNOS) is generally regarded as pro-inflammatory and cytotoxic. In diabetic myocardium, basal iNOS-levels are higher and the increase of iNOS-levels after IR injury is larger than in non-diabetic myocardium [30].

Third, SERCA_2a_ is crucial in postischemic myocardium by significantly decreasing intracellular Ca^2+^-overload [31]. It was shown before that SERCA_2a_-activity is depressed in ob/ob and DKO mice [8]. In the streptozotocin type I diabetic model, SERCA_2a_-expression after IR injury is increased versus WT animals. This is a possible explanation for the better preserved contractility after IR injury in this model [25].

Fourth, diabetic and metabolic syndrome models exhibit increased production of reactive oxygen species (ROS) [32]. ROS likely plays a central role in impairing mitochondrial energy metabolism, through mitochondrial uncoupling, but also through direct damage of mitochondrial components. This can lead to apoptosis [32].

Fifth, the activity of aldose reductase, a NADPH-dependent enzyme, increases during IR injury [33]. Since aldose reductase activity is already increased in diabetes [34], IR injury further reduces myocardial glycolysis and glucose oxidation and increases the glycosylation of advanced end-products. This leads to more extensive cross-linking of key structures within tissues and increased IR injury [34].

Sixth, chronic hyperglycemia leads to cardiac hypertrophy and decreased microvasculature density in myocardium. This microcirculation dysfunction causes increased vulnerability to IR [28].

Seventh, the exposure to abnormal substrates and cytokines makes the diabetic heart more prone to IR. It was recently shown that IL-33 is decreased in type I diabetic mice [28]. It is known that IL-33 prevents apoptosis, attenuates myocardial infarction and improves cardiac function. In this study was also shown that chronic activation of protein kinase C-β in diabetes contributes to myocardial dysfunction and infarct [28].

Finally, leptin deficiency leads to ventricular hypertrophy and heart failure [35,36]. McGaffin et al. reported that leptin has a protective role after IR [36]. Leptin deficient mice had worse survival, worse cardiac function and increased apoptosis, 4 weeks after IR injury. Leptin induces activation of signal transducer and activator of transcription-3, which induces transcription of several cardioprotective genes. These, in turn, inhibit reverse remodeling and apoptosis [35,36]. It is thus possible that leptin deficiency leads to reduced contractility and larger infarct size versus WT, independently from the other risk factors. Nevertheless, the effect of leptin deficiency during the acute phase of IR, as in our experiments, is not known. It is unlikely that STAT-3 activation plays a role during the first hours after IR injury, since transcription of new cardioprotective genes is required.

In hypothesis 1 and 2, we postulated that food restriction respectively ACE-I could reduce the effects of IR injury in mouse models of type II diabetes and the metabolic syndrome. In our study, 12 weeks food restriction or ACE-I, was not capable to preserve in vivo cardiac contractility, nor limit infarct size after IR injury. Nevertheless, ACE-I significantly improved load-independent contractility, as measured by PRSW, in the non-ischemic group.

In food restricted mice, the daily intake was limited to the amount of ‘normal’ free fed wild type mice, measured to be 2.5 g/day. After 12 weeks, the weight loss of ob/ob and DKO mice was 35 à 40% of initial body weight. We did find a slight but non-significant restoration of contractility after this treatment. In a previous study by our group [37], we found that food restriction with body weight reduction of 50 à 55%, partly restored contractility. So, it is possible that weight reduction of 35 to 40% is insufficient to significantly improve contractility in our mice models. Nevertheless, heart weight was reduced in ob/ob and even more in DKO, below the level of WT, demonstrating already the impact of the food restriction. A further explanation for the difference between the present and earlier studies is that the experimental conditions in the present study differed from the previous. In the study of Van den Bergh et al. [37], hemodynamic measurements were performed in closed chest mice. In this study, an IR protocol was investigated. This implicates that also in the control groups, left ventricular contractility was measured in the same experimental conditions as the ischemia-reperfusion group, i.e. with an open thorax and time matched.

Food restriction has been shown to reduce IR injury in wild type rodent models [13,38,39]. In renal IR injury, 30% food reduction leads to significant protection. The length of restriction, required for the onset of these renal benefits, is not known but also food restrictions, as little as 2 weeks, lead to significant protection. Interestingly, there is no increase in protection between 2 and 4 weeks of food restriction, suggesting that the maximal protection afforded by these short treatments was already reached after 2 weeks [38]. Also in the heart, different short-term (2 weeks) food restriction protocols, ranging from 10% to 70%, improve cardiac function following IR injury in wild type rats [39]. Yamagishi et al. [39] reported that 70% food restriction during 11 days, resulting in a weight reduction of 38% compared to free-fed rats, has a different effect on pre-ischemic contractility and sensitivity to IR injury. In this study, pre-ischemic cardiac function was reduced but cardiac recovery after IR injury was improved [39]. An improved ischemic tolerance via altered expression of functional proteins induced by low serum T3 levels, decreased coronary flow rate and change in metabolic flux, were suggested. Anaerobic glycolysis was more activated during ischemia in food restricted animals. This activation of glycolytic flux is fundamental to effective recovery of cardiac function during reperfusion after ischemia [39]. Nevertheless, the boundary between food restriction and under nutrition is weak. More than 70% food restriction results in depressed cardiac function [39]. The effect of food restriction on IR injury was never studied in the intended group, the obese type II diabetic patients and animal models. In our type II diabetic and metabolic syndrome mouse models, food restriction did not induce significant protection against IR injury.

The effects of food restriction and ACE-I on the previously described mechanisms of increased vulnerability of type II diabetic myocardium to IR injury are incompletely known. Data concerning the effects of these treatments on cardiac substrate utilization, energetics or aldose reductase activity are missing. Dyslipidemia seems to be a less important factor during IR injury, since infarct size and left ventricular contractility were not significantly different in DKO mice versus ob/ob. Although cholesterol-levels were normal after food restriction in ob/ob mice, this could not correct the increased sensitivity to IR injury.

ACE-I prevents downregulation of SERCA_2a _and phospholamban protein expression in heart failure [40]. Furthermore, ACE-I prevents the decrease in capillary density in type I diabetic rats [41]. This might explain the improved contractility in ob/ob and DKO mice with ACE-I.

It is widely accepted that food restriction and ACE-I reduce oxidative stress [13,14]. Nevertheless, nor food restriction nor ACE-I were capable to reduce IR injury in our mice models. This casts doubt on the role of oxidative stress in the increased vulnerability of diabetic myocardium to IR injury. No data are available concerning the effects of food restriction on capillary density, abnormal substrate utilisation or cytokinelevels in diabetic models.

Captopril has been reported to reduce infarct size in animal models, such as canine, porcine or rat models [16,17]. In the study of Itoh et al. [21], captopril did not protect WT mice against post-ischemic dysfunction and myocardial enzyme release. In contrast to this, a significant protection by captopril was found in mice with overexpression of p90 ribosomal S6 kinase. Since p90 ribosomal S6 kinase expression and activity are increased in diabetic mice, a protective effect of captopril was anticipated by Itoh et al. [21]. Nevertheless, we did not find infarct size reduction in our models of type II diabetes and the metabolic syndrome.

In this study, we investigated the effects of food restriction and ACE-I, because these treatments are standard therapies in type II diabetic and metabolic syndrome patients. Several other drugs with possible cardioprotective effects during IR injury have been developed. Peroxisome proliferator-activated receptor-γ agonists, such as rosiglitazone, have been shown to possess insulin-sensitizing and lipid-lowering and anti-inflammatory properties [42]. It has been demonstrated that peroxisome proliferator-activated receptor-γ agonists reduce IR injury in wild type and hypercholesterolemic models [42]. Nevertheless, it was shown by Ren et al. that the vasculoprotective effects of rosiglitazone are at least partially mediated by a reduction in local angiotensin II concentration [42]. Since ACE-inhibitors have similar effects on angiotensin II concentration and recent meta-analysis suggest that there is an increase in the risk of myocardial infarction, rosiglitazone was not investigated in this study [42].

Sulfonureas, such as metformin, are insulin-sensitizing agents, which reduce infarct size in non-diabetic animals by activation of cAMP-activated protein kinase and Akt. However, clinical and experimental studies in diabetic patients and models report a significantly higher cardiovascular risk and controversial effects on the effect on IR injury [26].

Recent evidence has indicated a possible importance of the endothelin system in the pathogenesis of diabetic complications [27]. Endothelin receptor blockade limits myocardial contractile depression in type I diabetic models [27]. The effect on IR injury was not studied. Since angiotensin II stimulates and bradykinin decreases endothelin-1 formation, it is conceivable that the majority of endothelin-1 formation is blocked by ACE-I. Furthermore, endothelin-receptor antagonism additional to ACE-I has no added benefit against cardiac dysfunction in type I diabetic rats [27].

Recently, Ichinomiya et al. demonstrated that high-dose fasudil, a Rho-kinase inhibitor, preserves postconditioning under acute hyperglycemic state in rats [43]. Rho-kinase activity is involved in diabetic endothelial dysfunction and regulation of Rho-kinase signaling is important for cellular function, such as contraction, mortality, proliferation and apoptosis. Correspondingly, Rho-kinase is known to be activated in ischemic myocardium. Inhibition of Rho-kinase activity is a matter of interest on myocardial protection against IR injury since it activates the ATP-sensitive potassium channels [43]. The effect of fasudil on postconditioning and IR injury in chronic hyperglycemic states, such as diabetes, has to be determined.

### Limitations

We used Captopril as ACE-I. Other ACE-inhibitors, with different lipid solubility and/or tissue specificity, might have affected the impact of IR injury. In this study, we investigated the acute effects of IR injury after an ischemia/reperfusion time of respectively 30 and 60 minutes. It is possible that the beneficial effects of these treatments appear in a later phase, for example during the remodeling phase.

The combination of food restriction and ACE-I was not investigated. In ob/ob mice, food restriction reduces weight and cholesterol levels and ACE-I reduces glycemia. It is possible that the combination of these effects improves myocardial damage. In DKO mice the additional effect is unlikely, since there was no reduction of cholesterol after diet and only moderate reduction of glycemia after ACE-I. Further studies have to be conducted to study these effects.

## Conclusion

Our mice models of type II diabetes and the metabolic syndrome are more sensitive to IR injury than their WT littermates, as they develop a significantly larger infarct size and worse in vivo cardiac contractility after 30 min ischemia followed by 60 min reperfusion. This mimics the clinical situation of patients with type II diabetes or the metabolic syndrome.

Twelve weeks of food restriction, with a weight reduction of 35 à 40%, did not alter left ventricular pre-ischemic contractility. Nevertheless, twelve weeks of ACE-I ameliorated contractility in the pre-ischemic ob/ob and DKO group significantly.

Food restriction and ACE-I did not correct the increased sensitivity for IR injury in our mice models of type II diabetes and the metabolic syndrome.

## Abbreviations

ACE: Angiotensin-converting enzyme; ACE-I: Angiotensin-converting enzyme inhibition; DKO: Double knock-out; E_a_ Arterial elastance; EDPVR: End-diastolic pressure volume relationship; Ees: End-systolic elastance; eNOS: Endothelial nitric oxide synthase; iNOS: Inducible nitric oxide synthase; IR: Ischemia-reperfusion; LAD: Left anterior descending artery; LDL: Low density lipoprotein; LDLR: Low density lipoprotein receptor; NO: Nitric oxide; Ob/ob: Leptin deficient mouse model for type II diabetes; P_ed_: End-diastolic pressure; PRSW: Preload recruitable stroke work; P_sys_: Systolic pressure; PV: Pressure-volume; ROS: Reactive oxygen species; SW: Stroke work; TTC: Triphenyl-tetrazolium chloride; WT: Wild type.

## Competing interest

The authors declare that they have no competing interests.

## Authors’ contribution

GVDM carried out the mice breeding, treatments, biochemical analysis, PV-loop experiments and infarct size determination, data and statistical analysis, and drafted the manuscript. IN, AV and WO contributed to mice breeding, treatments and biochemical analysis. WF participated in the design of the study and general supervision. PH designed the study, obtained funding, did supervision of the analysis and interpretation of data, and revised the manuscript for important intellectual content. All authors read and approved the final manuscript.
